# Extraskeletal Ewing sarcoma with uterine cornua attachment mimicking high grade endometrial stromal sarcoma: A case report and brief literature review

**DOI:** 10.1016/j.gore.2024.101537

**Published:** 2024-11-06

**Authors:** Wangpan Shi, Somaye Zare, Cheryl Saenz, Omonigho Aisagbonhi

**Affiliations:** a Department of Pathology, University of California San Diego, La Jolla, CA, USA; b Moores Cancer Center, University of California San Diego, La Jolla, CA, USA; c Department of Obstetrics, Gynecology and Reproductive Sciences, University of California San Diego, La Jolla, CA, USA

**Keywords:** Ewing, Uterus, Endometrial, Stromal, Sarcoma, Cancer

## Abstract

**Background:**

Ewing sarcoma is an EWSR1-rearranged aggressive malignancy that occurs commonly in bone and has small round blue cell morphology. A diagnostic challenge is presented in the cases of extraskeletal Ewing sarcoma involving solid organs, such as the uterus.

**Case report and brief literature review:**

We present the case of a 54-year-old female with a large pelvic mass connected to the uterine cornua and retroperitoneal soft tissue. Upon surgical debulking, the mass was characterized by infiltrative tumor cells with high nuclei-to-cytoplasmic ratio invading from the serosal surface into the myometrium. The initial diagnostic work-up was muddied by radiographic suggestion of uterine origin, histologic identification of uterine involvement and diffuse positivity for Cyclin D1, KIT and CD99, which raised the diagnostic possibility of high-grade endometrial stromal sarcoma. However, molecular testing that revealed *EWSR1::FLI* fusion, indicative of Ewing sarcoma. She was thus referred from the gynecologic oncology service to the soft tissue sarcoma service for adjuvant chemotherapy of vincristine/doxorubicin/cyclophosphamide and ifosfamide/etoposide (VDC/IE). We performed a literature review highlighting immunohistochemical and molecular features along with treatment and outcomes in case series of YWHAE-rearranged high-grade endometrial stromal sarcomas and Ewing sarcoma with uterine and/or retroperitoneal involvement.

**Conclusion:**

This case and accompanying literature review highlight the benefit of molecular testing in the context of round cell tumors involving the uterus and the importance of distinguishing high-grade uterine stromal sarcomas from sarcomas of soft tissue origin, as they have different treatment strategies.

## Highlights

• Ewing sarcoma involving the uterus morphologically overlaps with high-grade endometrial stromal sarcoma.
• Ewing sarcoma and high-grade endometrial stromal sarcoma are treated with different adjuvant chemotherapy.
• Molecular evaluation, with detection of EWSR1 rearrangement in Ewing sarcoma is critical for definite diagnosis.

## 1 Introduction

The first description of what is now known as Ewing sarcoma was in 1918 by Stout as a case of a tumor in the ulnar nerve with features of small and rounded cells forming rosettes resembling tissues from retinal gliomata (Stout, 1918). James Ewing described similar morphology of undifferentiated tumor cells in long bones three years later as diffuse endothelioma, now known as Ewing sarcoma (ES) (Ewing J, 1921). The majority of cases of Ewing sarcoma originate from the bone, however, the first study of extraskeletal Ewing sarcoma (EES) was reported by Angervall and Enzinger in 1975, describing 39 cases of solid, small, round tumor cells in soft tissue of the lower extremity that were morphologically indistinguishable from bone-based Ewing sarcoma (Angervall and Enzinger, 1975).

A diagnostic dilemma is presented when Ewing sarcoma involves the uterus, as primary uterine sarcomas may share similar histologic and immunohistochemical features. In particular, high-grade endometrial stromal sarcomas have round blue cell morphology, may have pseudo rosettes and are commonly positive for CD99, Cyclin D1 and KIT, all of which are also positive in Ewing sarcoma. Indeed, in a large case series of Ewing sarcoma of the female genital tract, three of 21 cases were initially misdiagnosed as high grade endometrial stromal sarcomas (Sharma et al., 2024).

We present a case of extraskeletal Ewing sarcoma with uterine cornua involvement initially diagnosed as high-grade endometrial stromal sarcoma due to morphologic and immunohistochemical overlap. We review the literature, focusing on case series with three or more cases reported, on Ewing sarcoma arising in or involving the uterus, and compare findings to those in high-grade endometrial stromal sarcomas. We emphasize the importance of molecular testing in distinguishing these two round blue cell tumors.

## 2 Case presentation

A 54-year-old female with a history of stage III breast invasive ductal carcinoma status-post mastectomy and chemoradiation four years prior, presented with urinary frequency and worsening right-sided pelvic pain, as well as weight loss.

**Fig. 1:**
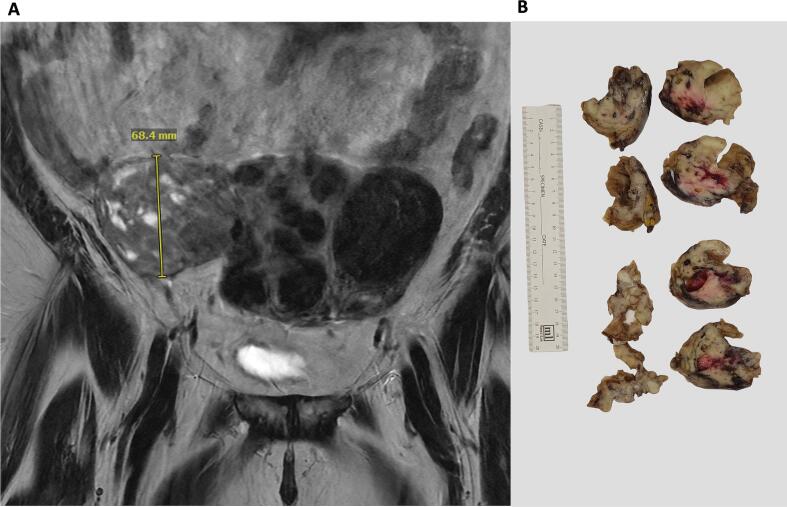
A: MRI revealed a fibroid uterus with a heterogeneous mass connected to uterine cornua and abutting surrounding soft tissue. B: Cut surface of the mass with solid and cystic components.

**Fig. 2:**
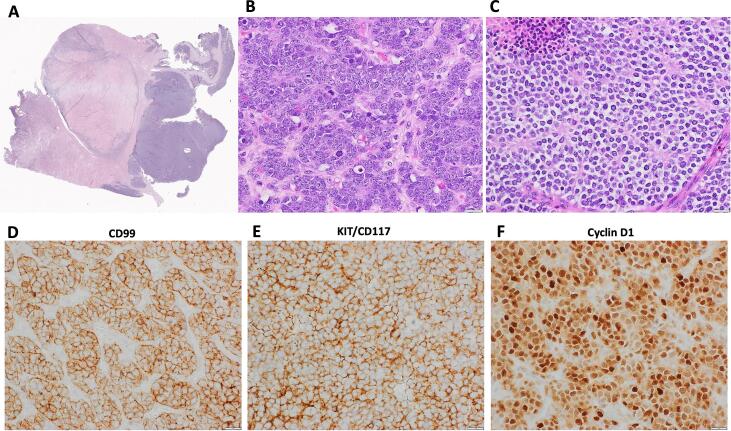
A: Scanning power of the mass showing invasion from the serosa to into the myometrium (0.5X). B: High power showing the tumor cells to be mitotically active with high nuclei/cytoplasm ratio and vesicular nuclei (40X). C: High power showing foci of rosette formation, necrosis and cytoplasmic clearing (40X). Immunohistochemical stains revealed the tumor cells to be positive for CD99 (D), KIT (E) and cyclinD1 (F).

She was found to have multiple fibroids with a heterogeneous lesion that measured 9.4 cm involving the right fundus/cornua of the uterus on MRI imaging (Fig. 1A). Intraoperatively, the mass was determined to be in the right retroperitoneal space, tracking along the broad ligament, and encasing the right external iliac artery. The mass was reported to be transected from the right uterine cornua. Gross evaluation of the specimen showed a detached, and fragmented ill-defined mass measuring 14 × 12 × 4 cm in aggregate. The cut surface of the mass showed both fleshy solid and cystic components with central hemorrhage and necrosis (Fig. 1B). Microscopically, the tumor invaded from the serosal surface into the myometrium (Fig. 2A), and showed sheets of tumor cells with pseudo rosette formation, small round hyperchromatic nuclei with high nuclei/cytoplasmic ratio, frequent mitosis (32 per 10 high power fields), foci with cytoplasmic clearing and necrosis (Fig. 2B–C). Immunohistochemical stains revealed the tumor cells to be diffusely positive for CD99, KIT, and cyclin D1 (Fig. 2D–F). The tumor cells were negative for other important markers including keratin, DOG1, synaptophysin, desmin, myogenin, WT-1, CD45, inhibin, GATA-3 and SOX-10 (Supplementary Table 1). Given the presence of a mass attached to the uterus and the diffuse positivity for cyclin D1 and KIT, a diagnosis of high-grade endometrial stromal sarcoma was initially favored. However, because of the retroperitoneal involvement, rosette formation by morphology and diffuse staining for CD99, Ewing sarcoma could not be excluded. The tumor was thus sent for Sarcoma Targeted Gene Fusion/Rearrangement Panel (SARCP) analysis (Supplementary Table 2), which identified the presence of *EWSR1::FLI1* fusion. As the mass had been intraoperatively described to be primarily located in the retroperitoneum, a diagnosis of retroperitoneal extra-skeletal Ewing sarcoma, with uterine involvement, was thus rendered. The patient was subsequently referred from the gynecologic oncology service to the soft tissue sarcoma service for adjuvant chemotherapy regimen of vincristine/doxorubicin/cyclophosphamide and ifosfamide/etoposide (VDC/IE) and is alive with disease (AWD) at four months post-surgery.

## 3 Discussion

Our patient presented with pelvic pain, urinary frequency and weight loss and was found to have a mass that involved both the uterus and retroperitoneum. Due to the non-specific origin and wide differential diagnostic possibilities that arose with the round cell morphology, the diagnostic work-up of this case was quite challenging. Tumors originating from the uterus and retroperitoneal soft tissue were the two main directions for diagnosis. One of the top differential diagnoses for retroperitoneal Ewing sarcoma includes desmoplastic small round cell tumor (DSRCT), which has clusters of round tumor cells surrounding a desmoplastic stroma. Ewing sarcoma can be misdiagnosed as DSRCT due to positive staining for keratin and/or synaptophysin (Trikalinos et al., 2021). Upon immunohistochemical studies, EMA, desmin, and WT-1 are characteristically positive in DSRCT, and they commonly show *EWSR1::WT1* translocation. Our case shared some morphologic overlap with the solid pattern of DSRCT with sheet-like monotonous cells. We excluded DSCRT based on the negative immunohistochemistry results for WT-1, desmin and EMA and the detection of *EWSR1::FLI1* fusion.

Another consideration in this location is dedifferentiated liposarcoma; it is usually a diagnostic consideration in any retroperitoneal tumor without a clear linage of differentiation by histomorphology and immunohistochemistry, but the presence of well-differentiated lipogenic area can be helpful. It is necessary, however, to identify MDM2 amplification by FISH, which in this case was non-amplified.

The uterine cornual connection presented a significant diagnostic challenge in this case. The morphology particularly raised the diagnostic possibility of high-grade endometrial stroma sarcoma. On microscopic examination, high-grade endometrial stromal sarcoma is characterized by primitive round cells with infiltrative growth. The nuclei of high-grade ESS harboring YWHAE-NUTM2A/B fusion are typically irregular, but they can also have monotonous small round cells with areas of rosette formation, similar to that seen in Ewing sarcoma (Lee et al., 2012).

The high-grade component of YWHAE-rearranged high-grade endometrial stromal sarcoma is typically diffusely positive for CD99, KIT, and cyclin D1; our review of YWHAE rearranged high-grade endometrial stromal sarcoma case series that also reported adjuvant chemotherapy regimen and/or outcome yielded 71 cases (Lee et al., 2012, Croce et al., 2013, Sciallis et al., 2014, Hemming et al., 2017, Alkanat et al., 2023, Kommoss et al., 2023); CD99 was positive in all three tested cases (100 %), KIT was positive in all four tested cases (100 %), and Cyclin D1 was positive in 12/15 tested cases (80 %) (Table 1). Ewing sarcomas generally show similar immunoprofile; upon review of reported immunohistochemistry in case series of EWSR1 rearranged uterine and retroperitoneal Ewing sarcomas (Sharma et al., 2024); (Sinkre et al., 2000, Cheng et al., 2021, Wei et al., 2024), 35 of 36 tested cases (97.2 %) were CD99 positive, 8 of 11 tested cases (72.7 %) were KIT positive and all three tested cases (100 %) were cyclin D1 positive (Table 1).

**Table 1:** Immunohistochemical and molecular features of reported case series of YWHAE rearranged high-grade endometrial stromal sarcoma and EWSR1 rearranged uterine and/or retroperitoneal Ewing sarcoma that also reported adjuvant chemotherapy regimen and/or outcome.

Study	Immunohistochemistry												Rearranged	Specific partner
	CD99	BCOR	FLI1	NKX 2.2	Keratin	KIT	Cyclin D1	Myogenin	Desmin	PR	ER	Syn		
**YWHAE-ESS**														
Lee 2012 (Lee et al., 2012)	N/A	N/A	N/A	N/A	− (4/13) N/A(9/13)	N/A	N/A	N/A	−(5/13)	+ in spindle areas (9/13)	+ in spindle areas (9/13)	N/A	YWHAE (13/13)	YWHAE-NUTM2A (2/13) YWHAE-NUTM2B (11/13)
Croce 2013 (Croce et al., 2013)	N/A	N/A	N/A	N/A	N/A	N/A	+(4/6) -(2/6)	N/A	N/A	−(5/6) +(1/6) spindle areas	−(6/6)	N/A	YWHAE (6/6)	YWHAE-NUTM2 (4/6)
Sciallis 2014 (Sciallis et al., 2014)	N/A	N/A	N/A	N/A	N/A	+moderate(4/4)	+(4/4)	N/A	N/A	−(4/4)	N/A	N/A	YWHAE (4/4)	N/A
Hemming 2017 (Hemming et al., 2017)	N/A	N/A	N/A	N/A	N/A	N/A	N/A	N/A	N/A	N/A	N/A	N/A	YWHAE (7/7)	N/A
Alkanat 2023 (Alkanat et al., 2023)	+ (3/3)	+ (3/4)	N/A	N/A	N/A	N/A	+(4/5)	N/A	N/A	N/A	N/A	N/A	YWHAE (5/5)	N/A
Kommoss 2023 (Kommoss et al., 2023)	N/A	N/A	N/A	N/A	N/A	N/A	N/A	N/A	N/A	N/A	N/A	N/A	YWHAE (36/36)	YWHAE-NUTM2 (33/36)
**EWING**														
Sinkre 2000 (Sinkre et al., 2000)	+(3/3)	N/A	N/A	N/A	+(1/3)	N/A	N/A	N/A	−(3/3)	N/A	N/A	−(3/3)	EWSR1	EWS-FL1(3/3)
Cheng 2021 (Cheng et al., 2021)	+(8/8)	N/A	N/A	N/A	+(4/8) -(4/8)	N/A	N/A	N/A	−(4/8) N/A(4/8)	N/A	N/A	+(7/8) -(1/8)	EWSR1 (8/8)	N/A
Sharma 2024 (Sharma et al., 2024)	+(12/12)	N/A	+(1/12) N/A(11/12)	+(3/12) N/A(6/12)	+(2/12) −(6/12) N/A(4/12)	+(1/12) N/A(11/12)	+(3/12) N/A(9/12)	−(2/12) N/A(10/12)	−(8/12) N/A(4/12)	−(3/12) N/A (9/12)	−(3/12) N/A (9/12)	+(2/12) −(2/12) N/A(8/12)	EWSR1 (12/12)	EWS-FLI1 (5/12) EWS-FEV (1/12) N/A (6/12)
Wei 2024 (Wei et al., 2024)	+(12/13)	N/A	+(7/13) −(3/13) N/A(3/13)	+(9/13) −(2/13) N/A(2/13)	+(4/13) −(8/13) N/A(1/13)	+(7/13) −(2/13) N/A(4/13)	N/A	N/A	N/A	N/A	N/A	+(7/13) N/A(6/13)	EWSR1 (12/13)	EWS-FLI1 (6/13)
Current case	+	N/A	N/A	N/A	−	+	+	−	−	−	−	−	EWSR1	EWS-FLI1

Thus, distinguishing Ewing sarcoma from high-grade endometrial stromal sarcoma is very challenging based solely on morphological features and immunohistochemical stains. However, this distinction is crucial because the post-operative adjuvant chemotherapy regimens are very different and may even involve different oncologic specialists. High-grade endometrial stromal sarcoma is managed by gynecologic oncologists, typically with doxorubicin-based chemotherapy regimens, similar to that used in uterine leiomyosarcomas (Cancer, 2025). Upon review of case series of YWHAE rearranged high-grade endometrial stromal sarcomas with reported adjuvant chemotherapy regimen and/or outcome, adriamycin/doxorubicin-based chemotherapy regimens were used in 5/10 (50 %) patients whose treatment regimens were reported (Supplementary Table 3). On the other hand, Ewing sarcomas are typically managed by bone and soft tissue sarcoma oncologists with vincristine/doxorubicin/cyclophosphamide and ifosfamide/etoposide (VDC/IE) regimens (Cancer, 2025). In our review of case series of EWSR1-rearranged uterine and retroperitoneal Ewing sarcomas, VDC or VDC/IE was used in 4 of the 9 patients (44 %) whose chemotherapy regimens were reported.

Furthermore, though both tumors are aggressive, Ewing sarcoma appears to be more aggressive than YWHAE-rearranged high-grade endometrial stromal sarcoma. Outcome data was reported in 62 of the 71 YWHAE-rearranged high-grade endometrial stromal sarcoma patients in the studies we reviewed – 25 of 62 (40.3 %) died of disease, 22 of 62 (35.5 %) were alive with disease and 15 of 62 (24.2 %) had no evidence of disease at a follow up interval that ranged from 4 to 3949 months (Supplementary Table 3). In contrast, of the 29 of 36 Ewing sarcoma patients with reported outcome data, 19 of 29 (65.5 %) died of disease, 3 of 29 (10.3 %) were alive with disease and 7 of 29 (24.2 %) had no evidence of disease at a follow up interval that ranged from 0.5 to 132 months (Supplementary Table 4).

Thus, molecular analyses such as FISH and NGS sequencing are of paramount importance in the diagnoses of round blue cell tumors arising in or involving the gynecologic tract. Furthermore, because the detection of certain fusions/rearrangements (such as NTRK and ALK) may warrant targeted therapy, molecular analyses with broad gene panels may be necessary.

## 4 Conclusion

We present a case of retroperitoneal extra-skeletal Ewing sarcoma with uterine attachment and histologic and immunohistochemical features overlapping with high-grade endometrial stromal sarcoma. We highlight the importance of molecular analyses for accurate diagnosis because the distinction between the two entities is vital for appropriate post-surgical adjuvant treatment.

## 5 Consent statement

Written informed consent is waived for this study according to institutional policy for case reports.

## Funding statement

This research received no specific grant from any funding agency in the public, commercial, or not-for-profit sectors.

## CRediT authorship contribution statement

**Wangpan Shi:** Writing – review & editing, Writing – original draft, Software, Resources, Methodology, Investigation, Formal analysis, Conceptualization. **Somaye Zare:** Writing – review & editing, Validation, Supervision, Methodology, Investigation. **Cheryl Saenz:** Writing – review & editing, Visualization, Supervision. **Omonigho Aisagbonhi:** Writing – review & editing, Writing – original draft, Visualization, Validation, Supervision, Methodology, Investigation.

## Declaration of competing interest

The authors declare that they have no known competing financial interests or personal relationships that could have appeared to influence the work reported in this paper.
