# Supplementary material for: Extraskeletal Ewing sarcoma with uterine cornua attachment mimicking high grade endometrial stromal sarcoma: A case report and brief literature review
Source: Gynecol Oncol Rep. 2024 Nov 6;56:101537. doi: 10.1016/j.gore.2024.101537 (PMC11705375; doi:10.1016/j.gore.2024.101537)
Supplement: Supplementary Data 1 [file mmc1.docx]

| Immunohistochemical stains | Results |
| --- | --- |
| CD99 | Positive |
| Cyclin D1 | Positive |
| CD117/KIT | Positive |
| DOG1 | Negative |
| Synaptophysin | Negative |
| Desmin | Negative |
| CD10 | Negative |
| WT-1 | Negative |
| PAX-5 | Negative |
| LCA/CD45 | Negative |
| Inhibin | Negative |
| CK5/6 | Negative |
| EMA | Negative |
| Estrogen receptor (ER) | Negative |
| Progesterone receptor (PR) | Negative |
| GATA-3 | Negative |
| HMB45 | Negative |
| E-cadherin | Negative |
| PAX-8 | Negative |
| Myogenin | Negative |
| Calretinin | Negative |
| Pancytokeratin | Negative |
| SOX-10 | Negative |
| PTEN | Retained |

Supplemental Table 1: Results of immunohistochemical studies performed in this case.

| **Gene Rearrangement** |  |  |  |
| --- | --- | --- | --- |
| **(5’Gene – 3’Gene)** | **(5’Gene – 3’Gene)** | **(5’Gene – 3’Gene)** | **(5’Gene – 3’Gene)** |
| *EWSR1-FLI1* | *WWTR1-CAMTA1* | *SFPQ-FOSB* | *EWSR1-PBX1* |
| *EWSR1-ERG* | *YAP1-TFE3* | *FUS-DDIT3* | *EWSR1-PBX3* |
| *EWSR1-FEV* | *CIC-DUX4* | *EWSR1-DDIT3* | *EWSR1-ZNF444* |
| *EWSR1-ETV1* | *CIC-FOXO4* | *EP400-PHF1* | *FUS-POU5F1* |
| *EWSR1-ETV4* | *CITED2-PRDM10* | *MEAF6-PHF1* | *FUS-KLF17* |
| *EWSR1-CREB1* | *BCOR-CCNB3* | *EPC1-PHF1* | *PDPN-PRKCB* |
| *FUS-FEV* | *BCOR-MAML3* | *MBTD-CXorf67* | *KIRREL-PRKCA* |
| *FUS-ERG* | *EWSR1-SMARCA5* | *EML4-ALK* | *LAMTOR1-PRKCD* |
| *SS18-SSX1* | *EWSR1-POU5F1* | *TPM3-ALK* | *NUMA1-SFMBT1* |
| *SS18-SSX2* | *EWSR1-NFATC2* | *TPM4-ALK* | *CD63 (RDH5)-PRKCD* |
| *SS18-SSX4* | *EWSR1-SP3* | *CLTC-ALK* | *SQSTM1-ALK* |
| *COL1A1-USP6* | *EWSR1-PATZ1 (ZSG)* | *RANBP2-ALK* | *VCL-ALK* |
| *SS18L1-SSX1* | *ZC3H7B-BCOR* | *CARS-ALK* | *ACTB-GLI1* |
| *PAX3-FOXO1* | *MED12-PRDM10* | *ATIC-ALK* | *GLI1-ACTB* |
| *PAX7-FOXO1* | *EWSR1-NR4A3* | *SEC31A-ALK* | *COL1A2-PLAG1* |
| *PRR12-FOXO1* | *TAF15-NR4A3* | *FN1-ALK* | *COL3A1-PLAG1* |
| *EWSR1-WT1* | *TCF12-NR4A3* | *PPFIBP1-ALK* | *RAB2A-PLAG1* |
| *HEY1-NCOA2* | *FUS-CREB3L2* | *RNF213 (ALO17)-ALK* | *HAS2-PLAG1* |
| *IRF2BP2-CDX1* | *FUS-CREB3L1* | *TRAF3-ALK (11-20)* | *HMGA2-LPP* |
| *NAB2-STAT6* | *EWSR1-CREB3L1* | *IGFBP5-ALK* | *LPP-HMGA2* |
| *CDH11-USP6* | *PAX3-MAML3* | *TFG-ALK* | *HMGA2-NFIB* |
| *MYH9-USP6* | *PAX3-NCOA1* | *FN1-FGFR1* | *HMGA2-PLPP3* |
| *SRSF3-USP6* | *PAX3-NCOA2* | *SRF-NCOA2* | *HMGA2-SETBP1* |
| *THRAP3-USP6* | *COL6A3-CSF1* | *TEAD1-NCOA2* | *C11orf95-MKL2* |
| *CNBP (ZNF9)-USP6* | *ZFP36-FOSB* | *VGLL2-NCOA2* | *TPR-NTRK1* |
| *OMD-USP6* | *FOSB-ZFP36* | *AHRR-NCOA2* | *TPM3-NTRK1* |
| *EWSR1-ATF1* | *WWTR1-FOSB* | *NCOA2-AHRR* | *BRD3-NUTM1* |
| *FUS-ATF1* | *SERPINE1-FOSB* | *EWSR1-CREB3L2* | *BRD4-NUTM1* |
| *ASPSCR1-TFE3* | *JAZF1-SUZ12* | *MIR143HG (CARMN)- NOTCH2* | *RAD51B-OPHN1* |
| *ETV6-NTRK3* | *JAZF1-PHF1* | *MIR143HG (CARMN)- NOTCH1* | *RAD51B-RRAGB* |
| *COL1A1-PDGFB* | *YWHAE-NUTM2B* | *NOTCH2-CEP128* | *DVL2-TFE3* |
| *CLTC-TFE3* |  |  |  |

Supplemental Table 2: Mayo Clinic Sarcoma Targeted Gene Fusion Panel.

| Study | Age (range, (years)) | No. cases | Location | Size  (range, (cm)) | Chemo | Radiation | Follow up | Duration (range, (months)) |
| --- | --- | --- | --- | --- | --- | --- | --- | --- |
|  |  |  |  |  |  |  |  |  |
| Sinkre, 2000^12^ | 47-71 | 3 | Uterus, corpus | 4.5-7.8 | Yes, NS (3/3) | Yes (1/3) | DOD (3/3) | 3-12 |
| Cheng, 2021^13^ | 13-47 | 8 | Uterus, cervix | 3.5-10.0 | Yes, NS (8/8) | Yes (6/8) | NED (1/8)  AWD (1/8)  DOD (5/8)  N/A (1/8) | 3-57 |
| Sharma, 2024^4^ | 15-61 | 12 | Uterus, cervix (4/12)  Uterus, corpus (6/12)  Uterus, anterior wall (1/12)  Uteropelvic (1/12) | N/A | N/A | N/A | NED (2/12)  AWD (1/12)  DOD (3/12)  N/A (6/12) | 0.5-132 |
| Wei, 2024^14^ | 16-70 | 13 | Retroperitoneal | 7.0-20.0 | Yes (9/13); VDC-IE (4/9) | N/A | NED (4/13)  AWD (1/13)  DOD (8/13) | 6-79 |
| Current case | 54 | 1 | Retroperitoneal with uterine attachment | 14.0 | Yes – VDC-IE | No | AWD | 4 |

Supplemental Table 3: Clinical features of reported case series of EWSR1 rearranged uterine and retroperitoneal Ewing sarcoma. Abbreviations: DOD-dead of disease, NED-no evidence of disease, AWD-alive with disease; VDC-IE: Vincristine, Doxorubicin, Cyclophosphamide alternating with Ifosfamide, Etoposide regimen; VAC-IE: Vincristine, adriamycin D and cyclophosphamide alternating with Ifosfamide, Etoposide regimen; NS – not specified; N/A – not available

| Study | Age  (mean) | No. | Extra-uterine extension | Tumor metastasis | Size | Chemo | Follow up | Month (mean) |
| --- | --- | --- | --- | --- | --- | --- | --- | --- |
|  |  |  |  |  |  |  |  |  |
| Lee 2012^6^ | 28-67 | 13 | + (4/13) | N/A | 6.4 | adriamycin and ifosfamide (1/13) | AWD (8/13) NED (1/13) DOD (2/13) N/A (3/13) | 12-108 |
| Croce 2013^7^ | 31-62 | 6 | + (1/6) | N/A | N/A | N/A | DOD (1/6)  NED (1/6) AWD (3/6) N/A (1/6) | 6-88.7 |
| Sciallis 2014^8^ | 45-52 | 4 | +( 4/4) | N/A | N/A | N/A | DOD (2/4) NED (1/4) AWD (1/4) | 24-150 |
| Hemming 2017^9^ | 42-47 | 7 | N/A | distant met (6/7) | N/A | doxorubicin (3/7) gemcitabine, docetaxel (1/7) AIM (2/7)  No (1/7) | DOD (3/7) NED (3/7) AWD (1/7) | 6-123 |
| Alkanat 2023^10^ | 22-50 | 5 | N/A | + (4/5) | N/A | Gemcitabine, docetaxel (1/5)  Doxorubicin (1/5)  N/A (3/5) | DOD (3/5)  N/A (2/5) | 4-34 |
| Kommoss, 2023^11^ | 16-89 | 36 | + (14/30) | N/A | N/A | N/A | NED (9/36)  AWD (9/36)  DOD (14/36)  N/A (4/36) | 55-3949 |
|  |  |  |  |  |  |  |  |  |

Supplemental Table 4: Clinical features of reported case series of YWHAE rearranged high-grade endometrial stromal sarcoma with reported adjuvant chemotherapy regimen and/or outcome. Abbreviations: DOD-dead of disease, NED-no evidence of disease, AWD-alive with disease; AIM: Adriamycin, Ifosfamide and Mesna; N/A – not available
